# Bio-assisted synthesis of bimetallic nanoparticles featuring antibacterial and photothermal properties for the removal of biofilms

**DOI:** 10.1186/s12951-021-01183-x

**Published:** 2021-12-28

**Authors:** Roman Nudelman, Shira Gavriely, Darya Bychenko, Michal Barzilay, Tamilla Gulakhmedova, Ehud Gazit, Shachar Richter

**Affiliations:** 1 Department of Materials Science and Engineering, Faculty of Engineering, Tel Aviv University, 69978 Tel-Aviv, Israel; 2 University Center for Nano Science and Nanotechnology, Tel-Aviv, Israel; 3 The Shmunis School of Biomedicine and Cancer Research, Faculty of Life Sciences, The George S. Wise, Tel-Aviv, Israel

**Keywords:** Antibacterial nanoparticles, Photothermal nanoparticles, Biofilms removal, Nanofibers, Jellyfish, Bio-assisted synthesis

## Abstract

Biofilms are responsible for about considerable amounts of cases of bacterial infections in humans. They are considered a major threat to transplant and chronic wounds patients due to their highly resistant nature against antibacterial materials and due to the limited types of techniques that can be applied to remove them. Here we demonstrate a successful in-situ bio-assisted synthesis of dual functionality nanoparticles composed of Silver and Gold. This is done using a jellyfish-based scaffold, an antibacterial material as the templating host in the synthesis. We further explore the scaffold’s antibacterial and photothermal properties against various gram-negative and positive model bacteria with and without photo-induced heating at the Near-IR regime. We show that when the scaffold is loaded with these bimetallic nanoparticles, it exhibits dual functionality: Its photothermal capabilities help to disrupt and remove bacterial colonies and mature biofilms, and its antibacterial properties prevent the regrowth of new biofilms.

**Supplementary Information:**

The online version contains supplementary material available at 10.1186/s12951-021-01183-x.

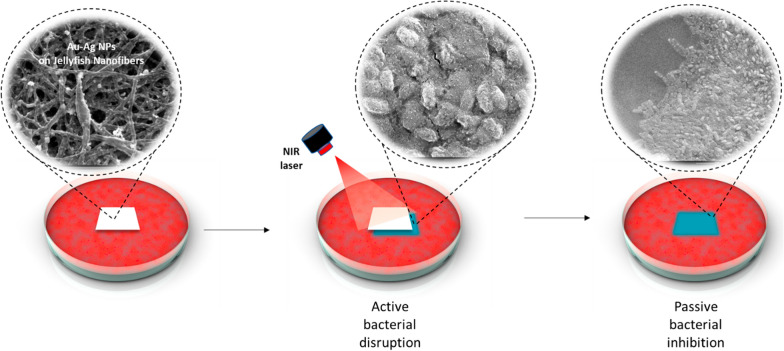

## Introduction

Biofilms (BFs) are composed of microorganisms such as bacteria that tend to irreversibly attach to wet surfaces to form a hydrated matrix of extracellular polymeric substance (EPS) [1, 2]. These colonies can naturally grow on various wet surfaces, including rocks, organic and inorganic materials, metal pipelines, heat exchangers, teeth, and various biomedical devices, thus presenting a significant adverse risk in a wide range of fields [3–5]. In this context, it was reported by the National Institute of Health that BFs are found in 80% of all known infections, thus causing considerable adverse economic and healthcare-related effects [6, 7].

Several strategies have been applied to prevent BFs formation and remove mature ones. These include the preemptive disinfection of surfaces [8] or chemical and physical disruption of already formed BFs structures followed by their mechanical removal [6, 9, 10].

Nevertheless, none of those schemes have shown high efficiency, as fully matured BFs exhibit high resistance capabilities, which reduce the effectiveness of the above-mentioned methods [8, 11]. Moreover, most BFs-removal methodologies cannot be applied to infected sensitive surfaces such as medical devices, implants, and wound-healing, limiting their use in biomedical use. Therefore, alternative methodologies are needed to be applied that fit these applications [12, 13].

One of the most useful antibacterial materials that might be harnessed for this task is Silver (Ag) in the form of nanoparticles (AgNPs) since these can disintegrate the bacterial membrane and its DNA. [14, 15] However, in fully matured BFs, AgNPs are less effective because these prevent the penetration of Ag ions into the BFs’s inner layers [16, 17].

It has been suggested that the application of heat in parallel with the application of the antimicrobial NPs might help to address this problem: the local heat applied induces sufficient thermal damage to the BF’s EPS allowing penetration of AgNPs that successfully damage the bacteria [18, 19]. A suitable biocompatible material that can exhibit photo-induced localized heat while also exhibiting a mild antibacterial effect is Gold (Au) in its various nanoparticle forms (AuNPs). For this reason, AuNPs have been extensively explored for their potential application in photothermal (PT) -based applications such as hyperthermal cancer therapy, wound healing, and biofilm control [20–22].

Thus, it can be hypothesized that using an appropriate scaffold in which AuNPs and AgNPs are embedded within, or preferably synthesized NPs composed of the two metals, is of great interest and might be used for efficient BFs removal [23–26]. In this respect, we recently reported on a non-woven biocompatible scaffold processed by the electrospinning (ES) method, allowing the *in-situ* one-pot spontaneous synthesis of NPs within [27]. The scaffold comprises Jellyfish nanofibers (JFNF) composed of JF’s collagen, Q-mucin protein, and polycaprolactone (PCL). In this respect, we have shown that in addition to its natural wound healing characteristics, JFNF can actively and spontaneously reduce Ag ions and form AgNPs directly on the nanofibers’ surface [27–29].

Here we take a step forward and report the successful in-situ synthesis of PT AuNPs, and bimetallic NPs composed of Au and Ag, using the JFNF as the templating host in the synthesis. We further explore the scaffold’s antibacterial and PT properties against various gram-negative and positive model bacteria with and without photo-induced heating at the Near-IR (NIR) regime (λ = 808 nm). Furthermore, we show that when the scaffold is loaded with these bimetallic NPs and placed on top of mature BF, it exhibits dual functionality: Its photothermal capabilities help to disrupt and remove bacterial colonies and mature biofilms, and its antibacterial properties prevent the regrowth of new BFs.

## Results and discussion

### JFNF: preparation and characterization

**Fig. 1:**
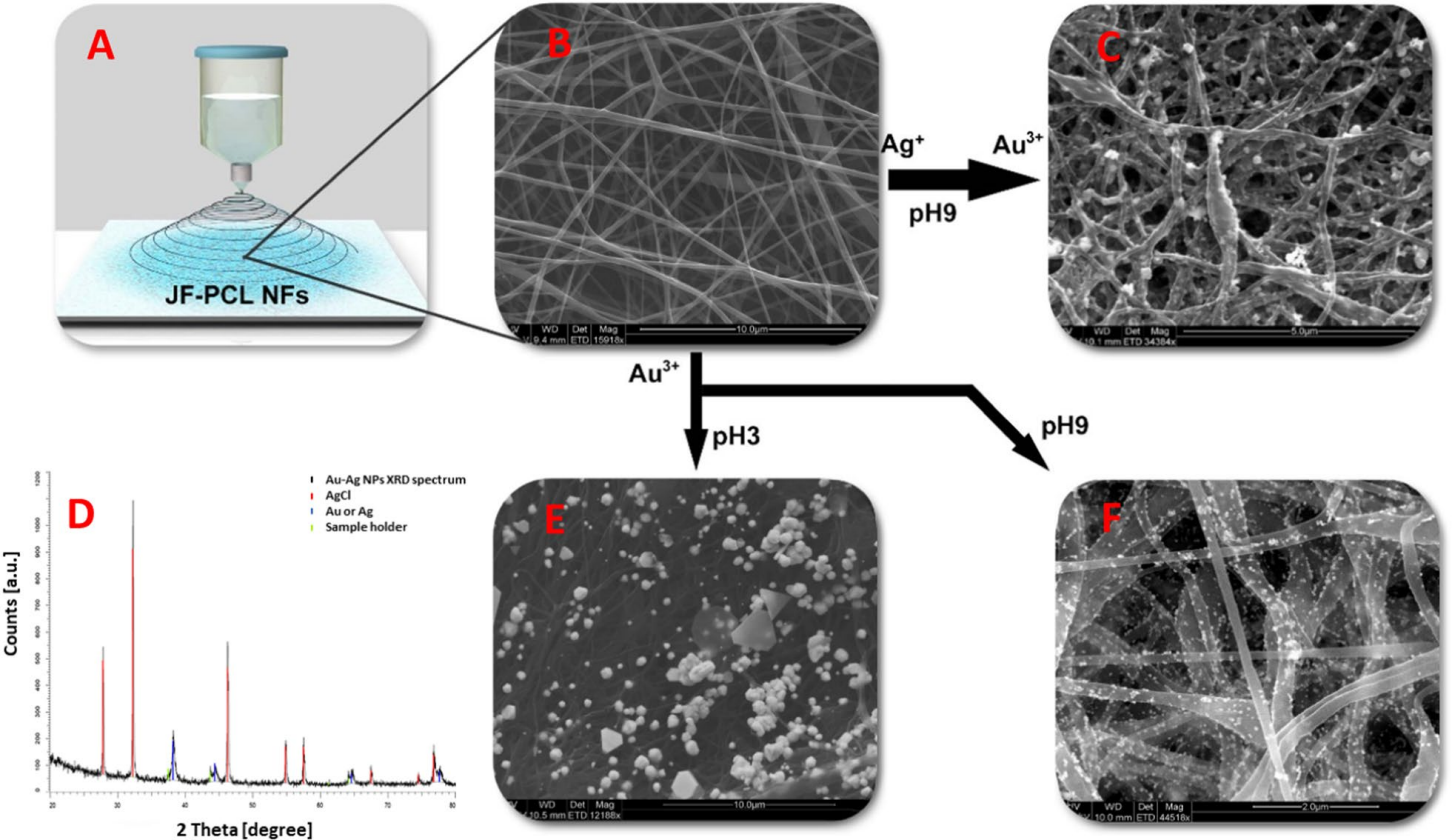
Scaffold preparation.** A** Schematic presentation of the ES process of JFNF nanofibers. ESEM images of** B** JFNF nanofibers,** C** Au–Ag NPs,** E** AuNPs, pH 3,** F** AuNPs, pH 9; all grown on JFNF scaffolds.** D** XRD spectrum of synthesized Au–Ag NPs. The peaks at 27.8°, 32.2°, 46.2° 54.9°and 57.6° correspond to the lattice planes of (111), (200), (221), (311), (222) respectively and represent the face-centered cubic (FCC) structure of AgCl crystal. The remaining peaks at 38.2°, 66.4°, and 77.2° are attributed to the lattice planes of (111), (220), and (311) also correspond FCC structure of Au–Ag NPs

Preparation of the JFNF was done using previously published protocol [27]. In short, locally collected jellyfish specimens (*Rhopilema nomadica*) were processed by mechanical cutting while the needed proteins collagen and Q-mucin were extracted by solvent precipitation and centrifugation. Next, JF proteins were dissolved and added to a PCL solution to prepare the ES solution used in a home-built electrospinning setup (Fig. 1A). Scaffold structure optimization was performed by varying the process parameters such as JF/PCL ratio, the voltage applied (13–17 kV), and tip-sample distance. Figure 1B shows the scaffold’s environmental scanning electron microscopy (ESEM), highlighting its fibrous nature. For additional analysis of the scaffold (See also Additional file 1: Figures S1 and S2).

### In situ synthesis of AuNP and Bimetallic NPs

As previously shown, this scaffold can be used to reduce Ag ions into AgNPs [27]. We attribute this phenomenon to the chemical reduction properties of Q-mucin glycoproteins’ presence on the fiber surface [30, 31]. Notably, as Silver’s electronegativity value is + 1.93 eV, it can be concluded that it is possible to synthesize other noble metal NPs that exhibit higher electronegativity values, including Au (+ 2.54 eV) [32] and bimetallic NPs.

Control over the synthesis products can be achieved via the pH of the reaction [33] because the latter affects the proteins’ ternary structure, which, in turn, determines the number of sites available for the reaction. To test this effect in our case, we synthesized AuNPs and bimetallic (Au–Ag) NPs complexes on the JFNF in pivotal acidic (pH 3) and alkaline (pH 9) environments. Figure 1C–F Shows Environmental Scanning Electron Microscopy (ESEM) images and corresponding X-ray diffraction (XRD) spectra of the scaffold. At pH 3, large spherical particles and hexagonal and triangular crystals were found (Fig. 1D, Additional file 1: Figure S3; Table S1), while small particles were generated at alkaline conditions. The phenomenon is attributed to the disulfide bonds protonation allowing the formation of a large area available for the synthesis at pH 3, resulting in large crystalline structures. In contrast, the disulfide bonds are deprotonated and tightly packed under alkaline conditions, limiting the volume and area of nucleation, resulting in small spherical AuNPs **(**Fig. 1E, Additional file 1: Figure S4; Table S1).

Next, we synthesized the bimetallic NPs. In this study, Ag and Au ions were added simultaneously to the scaffold in alkaline pH. Figure 1C depicts the ESEM images showing the synthesized particles. With the limitation of the characterization techniques, we hypothesize that the obtained structures are in the form of Au–Ag solid-solution as often obtained in the steady-state phase diagram [34]. XRD analysis (Fig. 1D and Additional file 1: Figure S5) of the bimetallic NPs clearly indicates the formation of Au, Ag, and AgCl crystals.

Correlation between the chemical reducing substrate (JFNF) properties and the size and structures of NPs reveals that larger fibers facilitated the growth of larger spherical particles than smaller-diameter ones (Table S1). The effect of fiber diameter on triangular and hexagonal Au particles’ growth is inconclusive due to the diverse particle population obtained. No correlation between the content of the solution and particle size was found (Additional file 1: Figure S6). Changing the pH didn’t affect the morphological structure of the JFNF and they retained their original structure.

### Photothermal properties of AuNPs and Ag-Au NPs

**Fig. 2:**
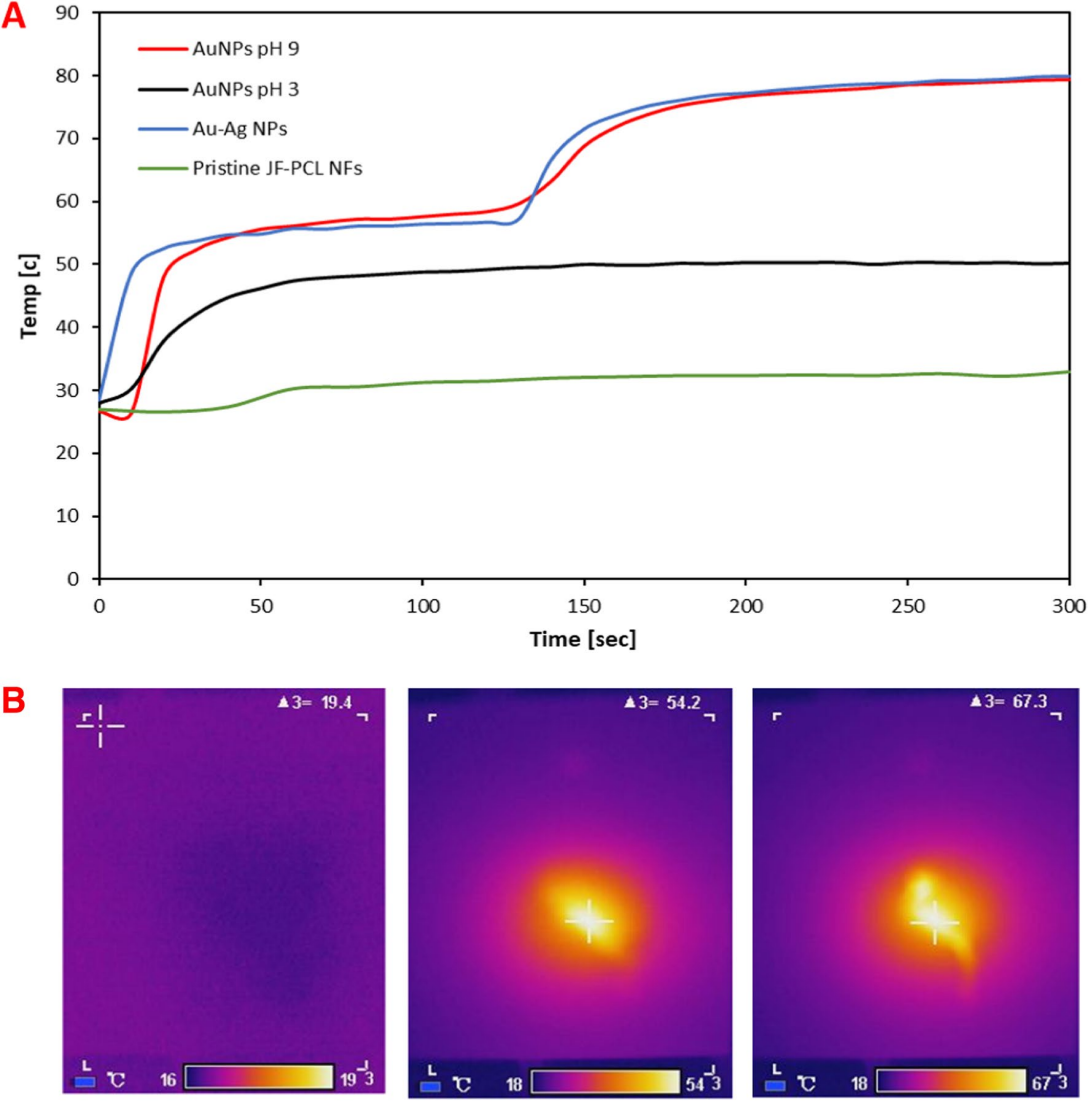
PT characterization. **A** Time-dependent measurements of AuNPs and Au–Ag NPs on JFNF scaffold synthesized at different pH. **B** Thermal images of the scaffold decorated with Au–Ag NPs taken at (left) t = 0, (middle) t = 60 s, and (right) t = 150 s

Figure 2 and Additional file 1: Figure S7 show the results of time-resolved PT measurements (excitation at λ = 808 nm) as a function of the synthesis pH, irradiation time, and laser power.

While it is evident that the pristine scaffold did not show PT characteristics, the NPs-doped scaffolds exhibited pronounced PT properties: AuNPs synthesized at pH 3 reached an elevated temperature of ~ 45 °C, while the ones synthesized at pH 9 reached a higher temperature of 80 °C. Notably, the bimetallic NPs produced at this pH showed a similar PT profile, indicating that these can function both as antibacterial and PT materials.

The time-dependent characteristics can be understood in light of thermogravimetric analysis (TGA, Additional file 1: Figure S8) of these materials, which shows a distinct melting point of the host scaffold around 60 °C, corresponding to the sharp increase in the PT profiles. This transition was not observed in AuNPs produced at pH 3 since the maximum heating temperature did not reach the melting point. The difference between the PT characteristics found in pH 3 and pH 9 can be attributed to the different size and dispersion of the particles produced in the syntheses (see also Additional file 1: Figures S3, S4; Table S1): It is known that light localization at hot spots between the dense population of particles, increases the amount of non-radiative processes which are transferred to heat [35]. Therefore, heat is generated more efficiently at the small and dense population (pH 9) than at the large and scattered ones (pH 3).

### Antibacterial and antibiofilm activity

The PT experiments allowed us to optimize and tune the conditions needed to be applied in the PT-induced antibacterial and antibiofilm experiments. In this respect, we choose irradiation times of ~ 60 s at moderate laser power (1 W/Cm^2^), which allows heating the scaffold to desired temperatures without damaging it.

**Fig. 3:**
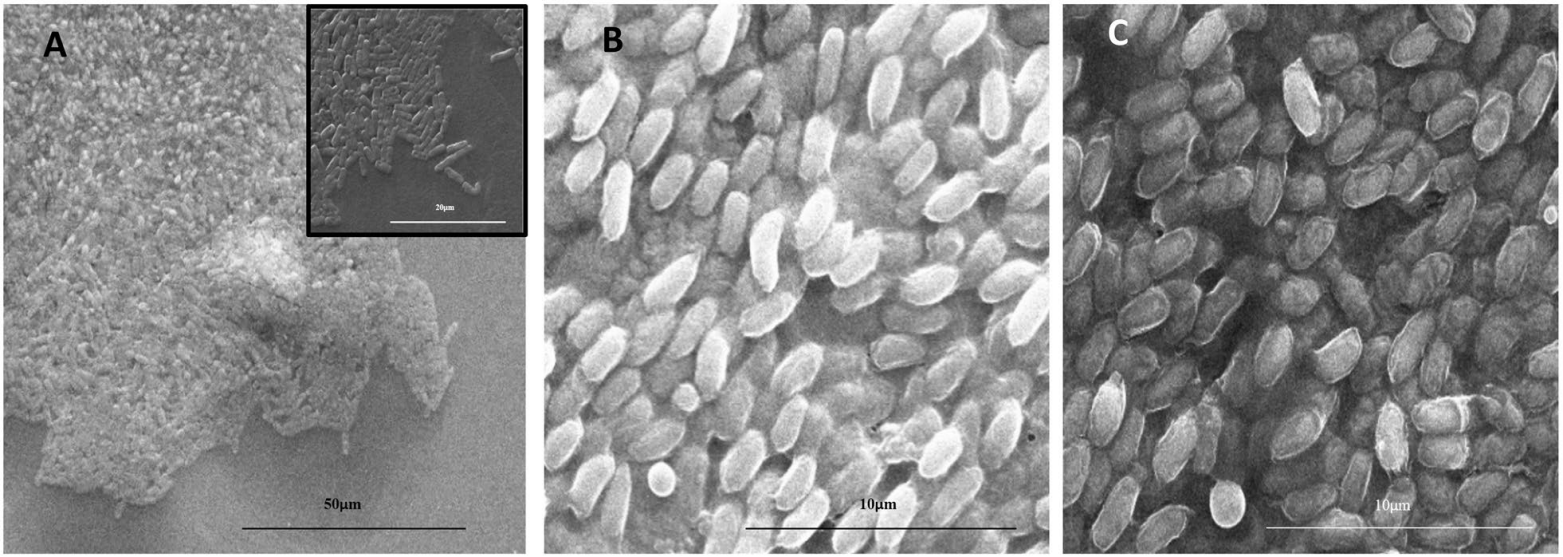
ESEM images of: **A** the area after the disk-diffusion test. The treated regime is clear of bacteria, while the bacteria at the periphery are present; inset: zoom-in of the borderline. **B** Bacteria cultures in the area that was not irradiated by laser. The bacteria colony remained alive. **C** The area underneath JFNF scaffold loaded with Au–Ag NPs after 60 s of laser irradiation. The damage to the bacteria’s EPS ‘s is clearly seen

To evaluate the scaffold’s antibacterial (“passive”) and PT-induced (“active”) properties, we performed disk diffusion antibacterial qualitative assays against gram-positive *Bacillus subtilis* bacteria known for their resistance to the harsh environment [36]. Additional file 1: Figure S9 shows the results of the assay tested with and without laser irradiation. Clear evidence of antibacterial activity is shown in scaffolds with Au–Ag NPs. ESEM scan of the inhibition zone highlights the borderline between the treated area, which clearly became bacteria-free, and the untreated one (Fig. 3A).

Next, the antibiofilm properties of scaffolds with Au–Ag NPs were evaluated. In this case, due to the high resistance properties of the matured biofilms, we also applied PT treatment.

**Fig. 4:**
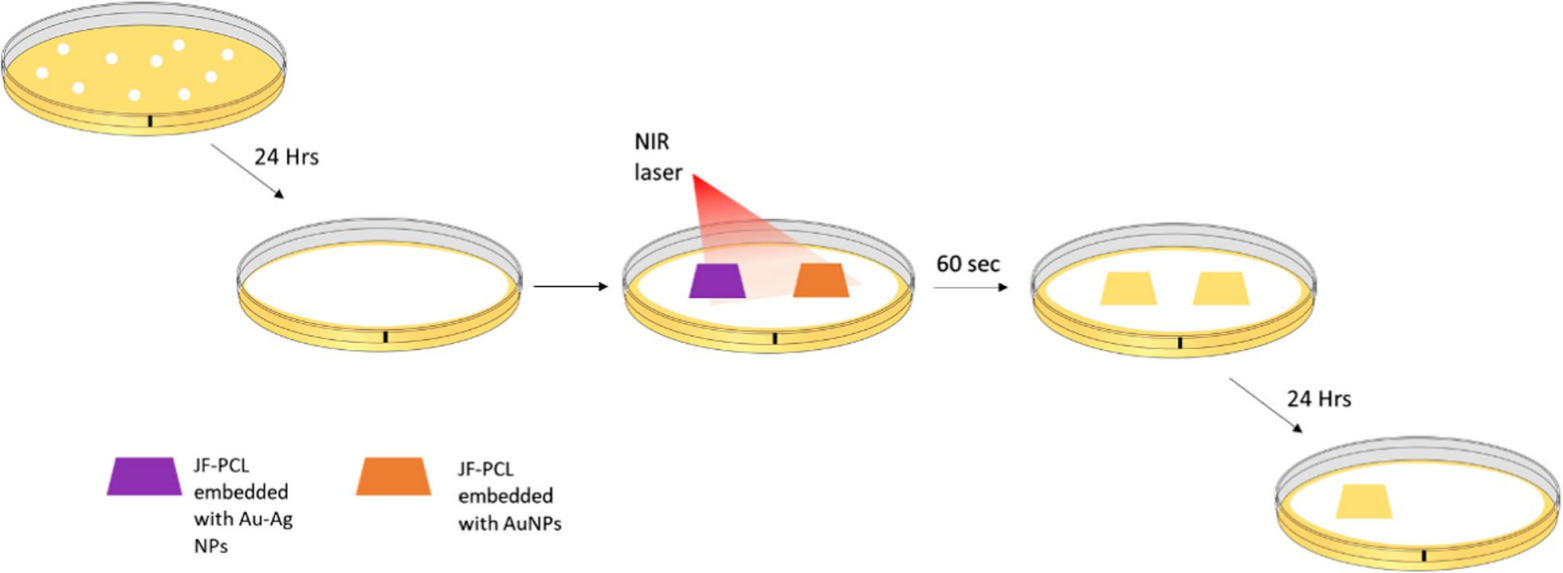
Schematic process of both passive and active multifunctional properties of Au–Ag JFNF scaffold. Scaffolds containing Au and Au–Ag NPs were put on a mature biofilm surface and irradiated by 808 nm laser. The scaffolds showed complete disruption of the biofilms. After 24 h, regrowth of the biofilm was observed only in the case of the Au-based scaffold, while no growth of the biofilm was observed in the case of the Au–Ag-based scaffold

It is evident that upon irradiation (Fig. 3 and Additional file 1: Figure S9), mature biofilms located directly underneath the NP-decorated scaffolds were denatured. The corresponding ESEM images indicated that the produced thermal shock had caused massive damage to the bacteria’s membrane, causing membrane collapse (Fig. 3B, C). Due to that short lasing time, it can be concluded that the destruction of the biofilm takes place solely because of the high-temperature increment and not due to the passive antibacterial effect, which occurs at longer time scales. However, we have found that the irradiated bimetallic-based scaffolds kept the treated surface clean of biofilms for at least 24 h after removal in contrast to the AuNPs-based scaffold in which regrowth of colonies was found (Fig. 4 and Additional file 1: Figure S10). We attribute this observation to the diffusion of the antibacterial silver ions from the NPs to the surface, making this surface resistant to bacterial regrowth.

Laser irradiation applied to a reference sample composed of a pristine scaffold did not affect the bacterial colony. Interestingly, this type of scaffold exhibited strong adhesion to the biofilm, causing partial biofilm removal from the growth medium to the scaffold without the need for heating (Additional file 1: Figure S10).

### Quantitative antibacterial growth inhibition assay

**Fig. 5:**
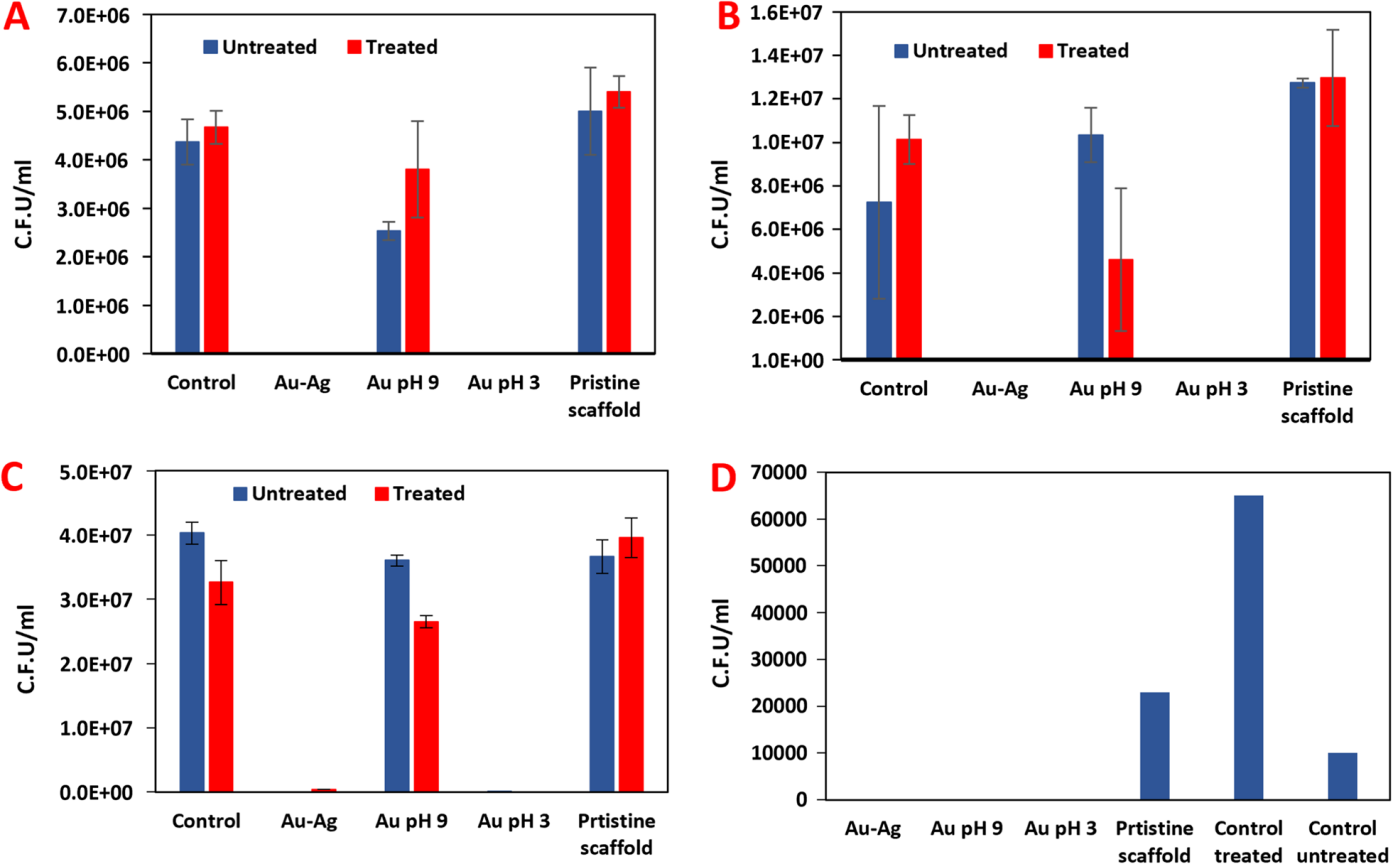
**A**–**C** Colony-Forming Unit (CFU) counts of bacteria approximately 24 h after addition of the scaffolds composed of bacterial growth substrate (“control “), bimetallic NPs (“Au–Ag “), AuNP synthesized at pH9, at pH3, and of a scaffold without NPs (“pristine scaffold “). The graphs present results acquired with and without laser irradiation (“treated “ and untreated “, repectively), performed on *E. coli* (**A**), *S. epidermidis* (**B**) and *P. aeruginosa* (**C**) assays.** D** CFU analysis of *P. aeruginosa* biofilm. All samples that contained NPs showed destruction of the BFs underneath them and bacterial death compared to the controls

To check the validity of our methodology to different types of bacteria, we performed a quantitative antibacterial growth inhibition assay in aqueous media. This was done using three different bacterial strains: *E. coli* (Gram-negative) (Fig. 5A), *S. epidermidis* (Gram-positive) (Fig. 5B), and *P. aeruginosa* (Gram-negative, Fig. 5C), a pathogenic bacterium capable of forming biofilm layers, as a model bacterial strain.

The analyses indicate that the growth of all three tested bacterial strains was utterly inhibited by scaffolds decorated with Au–Ag NP and AuNPs (pH 3), and this, with and without application of laser. These observations suggest that the scaffold’s passive antibacterial activity exhibits high antibacterial properties, and laser irradiation is not required to suppress bacterial growth in these cases, as the difference between the two in each scaffold was not significant.

Surprisingly, despite their excellent PT properties, AuNPs (pH 9)-based scaffold showed no significant inhibition with or without the laser irradiation except in the case of *S. epidermidis* (Fig. 5B). This can be attributed to the slow diffusion of metal ions and short irradiation time, which was inefficient in damaging the bacteria in this particular case. In all cases, pristine fibers scaffold without the Np did not show any antibacterial effect with and without laser irradiation.

### Quantitative antibiofilm inhibition assay

Finally, we performed quantitative analysis on mature *P. aeruginosa* BFs (Fig. 5D). It is evident that scaffolds that contain NPs successfully devastated the BF. The heat generated by the irradiated Au–Ag NP and AuNP (pH 3 & pH 9) was sufficient to completely denaturate the biofilm beneath the scaffolds even in a short irradiation time of 30 s by this “sanitizing” the solid growth medium. In this case, the passive antibacterial function of the scaffolds is not necessary for bacterial removal but is nevertheless very useful for the prevention of new contaminations as demonstrated in the qualitative assay which was performed on *Bacillus subtilis* biofilm (Fig. 3, Additional file 1: Figure S11).

Reference experiments performed on pristine JFNF scaffold (Fig. 5A–C “pristine scaffold”) showed no significant antibacterial activity, with or without laser treatment. In addition, direct irradiation by laser on the bacteria or biofilm did not show any inhibition growth or biofilm removal.

## Conclusions

In summary, the synthesis and the characteristics of a novel anti-biofilm scaffold is demonstrated. The scaffold is comprised of electrospun Jellyfish-based nanofibers decorated with bimetallic nanoparticles. The dual functionality of the scaffold was evaluated via its photothermal and antibacterial properties. We leverage these properties and demonstrate how this scaffold can successfully disrupt, remove and eliminate the regrowth of mature biofilms colonies. We believe that this novel scaffold can be used for advanced treatments in many medical applications such as wound dressing, infections, and sensitive infected areas.

## Experimental section and methods

### Materials

Hydrogen tetrachloroaurate hydrate; 99.9%, Au 49%- (HAuCl_4∙3H_2_ O) was purchased from Sigma-Aldrich. Silver nitrate 99.999% (AgNO3); Glycine, and sodium hydroxide (NaOH) were all purchased from Sigma-Aldrich.

### JFNF Production

JFNF were prepared in the same manner as we previously described in ref [22]. Shortly, different w%/w% solutions of JF-Polycaprolactone (PCL)/AcOH solutions were electrospun under 12–17 kV voltage with a controlled flow rate of 2–3 µl/min, a distance of 25 cm in RT, and 70% humidity.

### Synthesis of AuNPs

A 1 cm × 1 cm piece of JFNF was added to 3 ml HAuCl $\bullet$ 4∙3H_2 O 2.5 mM in a 20 ml vial. The vial was covered with an aluminum sheet and was stirred for 20 min. Next, 2 ml of glycine buffer of pH 3 or 9 was added, and nitrogen gas was injected into the vial. The synthesis was kept stirred for six days. After the reaction completion, the NFs scaffold was washed with water and dried in the fume hood.

### Synthesis of Au–Ag NPs complex

A 1 cm × 1 cm piece of NF was added to 3 ml HAuCl_4∙3H_2 O 2.5 mM in a 20 ml vial and stirred for 20 min. After the initial stirring, 2 ml of glycine buffer pH 9 was added along with 0.25 ml of 0.1 M AgNO_3_ and nitrogen gas injection. The vial was covered with aluminum and kept stirred for six days. After the reaction completion, the NFs scaffold was washed with water and dried in a fume hood.

### Characterizations

ESEM imaging was carried out with the Quanta 200 FEG Environmental scanning electron microscope with an operational voltage of 20 kV. Sputtering of 10 nm gold was required prior to ESEM imaging for contrast enhancement. The Spattering was performed with the SPI sputter at1.2 kV for 120 s.

X-ray diffraction was performed by initial sample preparation, which included centrifugation of prepared Au and Ag NPs at 1000 rpm for 5 min, and further drying of the particles under vacuum.

Thermal analysis and confocal microscopy were employed to determine the thermal properties and the chemical composition of the NFs scaffolds. These techniques are elaborately explained in the SI section.

### Photothermal laser test

For the PT measurement, a home-built system consists of NIR laser (Laser Power Supply, model no. PSU-H-LED, 808 nm, By Changchun New Industries Optoelectronics Tech. co., LTD.) coupled with a thermal camera and confined in an optically sealed box. Different samples (slightly wet with dH_2_ O) of JFNF scaffolds with Au- and AgNPs were put on glass slides and irradiated with an IR beam. The temperature of the scaffold was measured by a thermal camera (IR-CAM-160 by MRC). The samples were irradiated for 5 min with 1 W.

### Disk diffusion antibacterial essay

Nutrient agar plates from a solution of Luria agar base were prepared, to create a substrate for bacteria growth. A 40μL sample of bacteria was plated on each substrates and then heated on 37℃ for 10 min (upside down). The substrates were supplemented with NFs scaffolds covered with silver/gold NPs. The substrates were incubated for 24 h in the oven at 37℃, as well as NPs-free plate and the commercial pad was used as a control. The area of clear solution underneath the NFs scaffolds was examined on each of the plates (cloudy areas indicate the growth of bacteria colonies).

### Laser test with Bacillus subtilis p479 biofilm

NFs scaffolds with Gold and Silver NPs were put on cultures of bacteria (the same procedure as described under “Antibacterial test”), and were radiated by IR beam for 60 s. The scaffolds were removed, and the cultures were put under the gauze pad soaked with glutaraldehyde (25%) for an hour to absorb the vapors (for the preservation of the bacteria). Then the petri dishes were put in a desiccator for dehydration for few days.

### Bacterial growth inhibition assay

Escherichia coli (K-12 strain, WT) bacteria were grown overnight in M9 minimal media and diluted 500-fold in M9 media. Staphylococcus epidermidis (ATCC 12228) bacteria were grown overnight in LB media and diluted 1,000-fold in LB media. Pseudomonas aeruginosa (ATCC 27853) bacteria were grown overnight in LB media and diluted 1,000-fold in LB media. Samples were placed in 2 Corning (3879) 96-well plates (Sigma-Aldrich, Israel), three repetitions per plate for each bacteria. 100 µl volumes of growth medium containing bacteria (5 × 10̂6 CFU/ml) were added to each well. Post treatment, the plates were covered with Breathe-Easy sealing membrane (Sigma-Aldrich, Israel) and incubated at 37 °C overnight. Growth inhibition was evaluated by CFU-count. Bacteria marked as control were grown solely in bacterial media, without any alterations.

## Supplementary Information

**Additional file 1: Figure S1**. (a) A confocal microscope image of 25/75 JF/PCL NFs (applied voltage 13kV) labeled with DTAF (x20 magnitude). (b) A confocal microscope image of 75/25 JF/PCL NFs (applied voltage 13kV) labeled with DTAF (x20 magnitude). (c) A column graph which displays the difference between the NFs intensities (x10 magnitude) as a function of the applied voltage and JF/PCL ratio. **Figure S2**. Mechanical properties of JF/PCL scaffolds (A) Young's modulus as a function of JF/PCL ratio. (B) Column graph which displays the difference between the NFs thickness as a function of the applied voltage and JF/PCL ratio. (C) Column graph which displays the difference between the NFs porosity Stress-strain curve as a function of JF/PCL ratio for (D) 13kV and (E) 17kV as a function of the applied voltage and JF/PCL ratio. **Figure S3**. AuNPs synthesized with pH 3 on different JF/PCL NFs scaffolds. (a) 25/75 (b) 33/66 (c) 50/50 (d) 66/33 (e) 75/25. **Figure S4**. AuNPs synthesized with pH 9 on different JF/PCL NFs scaffolds. (a) 25/75 (b) 33/66 (c) 50/50 (d) 66/33 (e) 75/25. **Figure S5**. XRD of AuNPs in pH 3 and pH 9. Inset is XRD of pristine JF/PCL scaffold. **Figure S6**. Particle size dependence on JF/PCL component ratio. **Figure S7**. Au-Ag NPs on NFs scaffold under different power output of 808nm laser. **Figure S8**. (a) DTA curves of 66/33 and 33/66 JF/PCL scaffolds. (b) TGA curves of 66/33 and 33/66 JF/PCL scaffolds. **Figure S9**. Disk diffusion test of Au NPs pH 9, AgNPs, Au-Ag NPs on NFS scaffolds, after 24 hours on a bacteria culture. (A) Bacteria substrates before scaffolds were removed. (B) bacteria substrates after scaffolds were removed. Au-Ag NPS (NFS scaffolds) that were put on bacteria culture (C) before laser NIR laser irradiation. (D) after 60 seconds of laser irradiation. (G) in area underneath NFS scaffold (50/50 13kv) with Au and Ag after 60 seconds of irradiation. **Figure S10**. (a) Area underneath pristine NFs scaffold (without NPs) after irradiation with laser for 60 sec. (b) ESEM image of the scaffold after irradiation. **Figure S11**. (a) Swab sample that were taken underneath scaffolds after laser irradiation. (b) Swab sample that were taken underneath the same scaffolds after incubation for 24 hours. **Table S1**. T-Test for AuNPs size grown in different conditions- pH and JF/PCL scaffold

## Data Availability

Not applicable.
