# Supplementary material for: Bio-assisted synthesis of bimetallic nanoparticles featuring antibacterial and photothermal properties for the removal of biofilms
Source: J Nanobiotechnology. 2021 Dec 28;19:452. doi: 10.1186/s12951-021-01183-x (PMC8715638; doi:10.1186/s12951-021-01183-x)
Supplement: Supplementary file 1 — Additional file 1: Figure S1. (a) A confocal microscope image of 25/75 JF/PCL NFs (applied voltage 13kV) labeled with DTAF (x20 magnitude). (b) A confocal microscope image of 75/25 JF/PCL NFs (applied voltage 13kV) labeled with DTAF (x20 magnitude). (c) A column graph which displays the difference between the NFs intensities (x10 magnitude) as a function of the applied voltage and JF/PCL ratio. Figure S2. Mechanical properties of JF/PCL scaffolds (A) Young's modulus as a function of JF/PCL ratio. (B) Column graph which displays the difference between the NFs thickness as a function of the applied voltage and JF/PCL ratio. (C) Column graph which displays the difference between the NFs porosity Stress-strain curve as a function of JF/PCL ratio for (D) 13kV and (E) 17kV as a function of the applied voltage and JF/PCL ratio. Figure S3. AuNPs synthesized with pH 3 on different JF/PCL NFs scaffolds. (a) 25/75 (b) 33/66 (c) 50/50 (d) 66/33 (e) 75/25. Figure S4. AuNPs synthesized with pH 9 on different JF/PCL NFs scaffolds. (a) 25/75 (b) 33/66 (c) 50/50 (d) 66/33 (e) 75/25. Figure S5. XRD of AuNPs in pH 3 and pH 9. Inset is XRD of pristine JF/PCL scaffold. Figure S6. Particle size dependence on JF/PCL component ratio. Figure S7. Au-Ag NPs on NFs scaffold under different power output of 808nm laser. Figure S8. (a) DTA curves of 66/33 and 33/66 JF/PCL scaffolds. (b) TGA curves of 66/33 and 33/66 JF/PCL scaffolds. Figure S9. Disk diffusion test of Au NPs pH 9, AgNPs, Au-Ag NPs on NFS scaffolds, after 24 hours on a bacteria culture. (A) Bacteria substrates before scaffolds were removed. (B) bacteria substrates after scaffolds were removed. Au-Ag NPS (NFS scaffolds) that were put on bacteria culture (C) before laser NIR laser irradiation. (D) after 60 seconds of laser irradiation. (G) in area underneath NFS scaffold (50/50 13kv) with Au and Ag after 60 seconds of irradiation. Figure S10. (a) Area underneath pristine NFs scaffold (without NPs) after irradiation with laser for 60 [file 12951_2021_1183_MOESM1_ESM.docx]

**Supplementary information**

Bio-assisted synthesis of bimetallic nanoparticles featuring antibacterial and photothermal properties for the removal of biofilms

Roman Nudelman^1,2^ Shira Gavriely^1,2^ Dasha Bychenko,^2,3^ Michal Barzilay,1,2 Tamilla Gulakhmedova^1,2^, Ehud Gazit,^2,3^, Shachar Richter^1,2*^

^1^Department of Materials Science and Engineering, Faculty of Engineering

^2^University Center for Nano Science and  Nanotechnology

^3^The Shmunis School of Biomedicine and Cancer Research, The George S. Wise Faculty of Life Sciences

Tel Aviv University, Tel-Aviv, 69978, Israel

E-mail: srichter@tauex.tau.ac.il


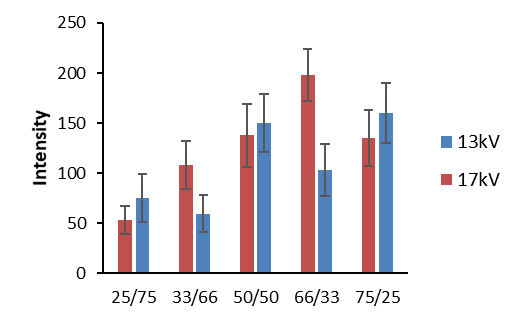

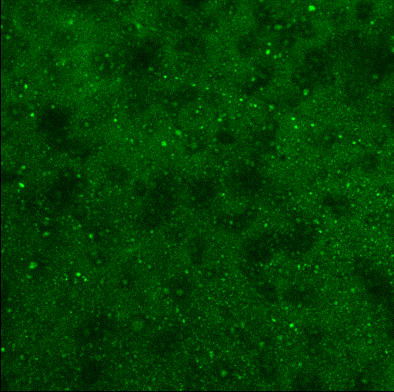


A


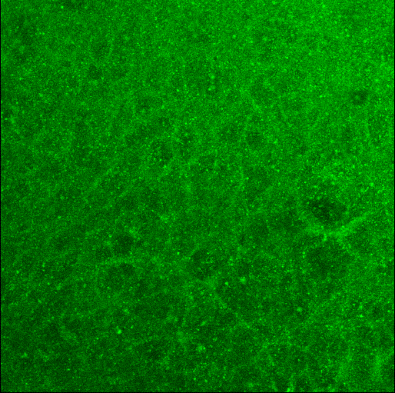


B

C

**Figure S1. (a) A confocal microscope image of 25/75 JF/PCL NFs (applied voltage 13kV) labeled with DTAF (x20 magnitude). (b) A confocal microscope image of 75/25 JF/PCL NFs (applied voltage 13kV) labeled with DTAF (x20 magnitude). (c) A column graph which displays the difference between the NFs intensities (x10 magnitude) as a function of the applied voltage and JF/PCL ratio.**

**
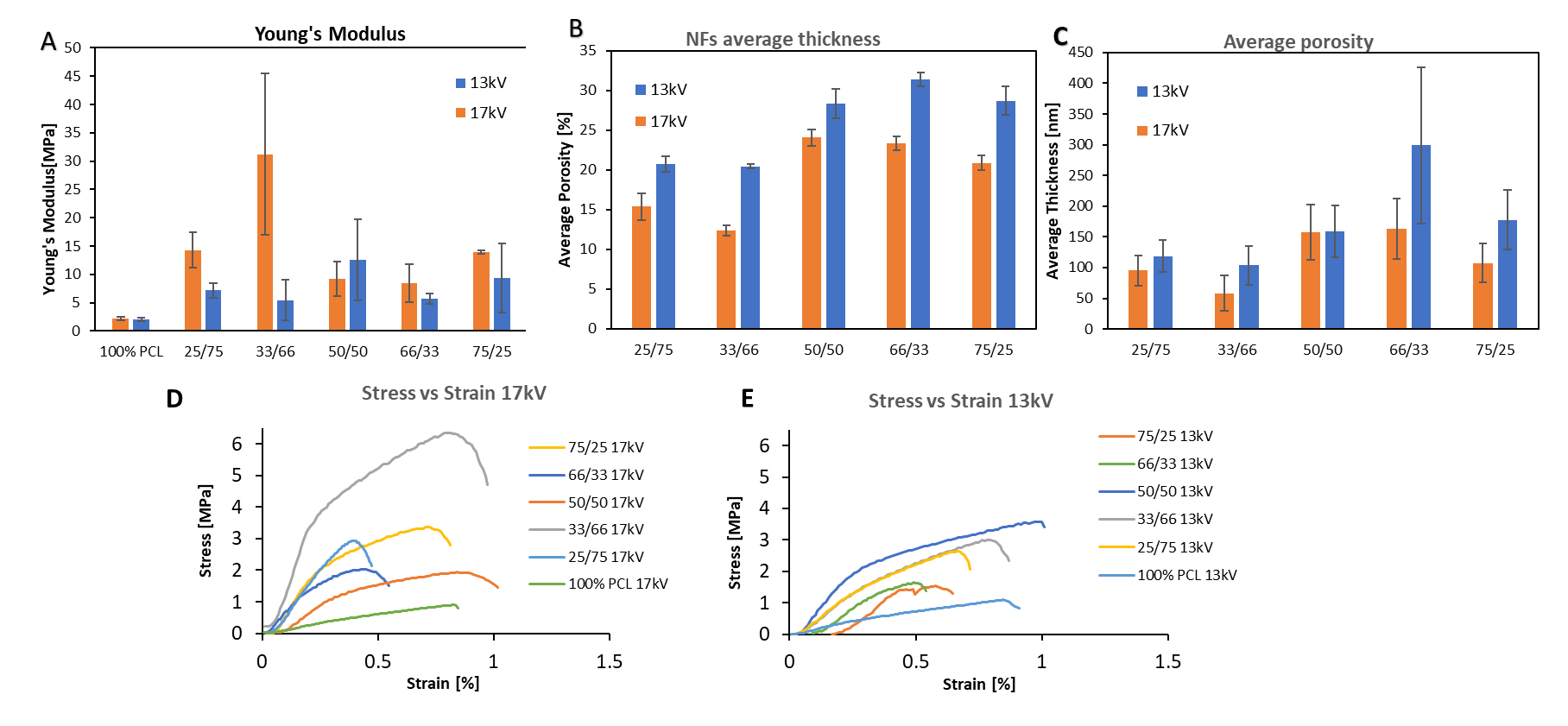
**

Figure S2. Mechanical properties of JF/PCL scaffolds (A) Young's modulus as a function of JF/PCL ratio. (B) Column graph which displays the difference between the NFs thickness as a function of the applied voltage and JF/PCL ratio. (C) Column graph which displays the difference between the NFs porosity as a function of the applied voltage and JF/PCL ratio.Stress-strain curve as a function of JF/PCL ratio for (D) 13kV and (E) 17kV

It is evident that reducing the scaffold's NF diameter takes place in higher applied voltages while the porosity decreases. This is attributed to higher electric forces applied to the solution [xx], resulting in the rapid formation of thin NF. No correlation between the content of the solution and these properties was found.


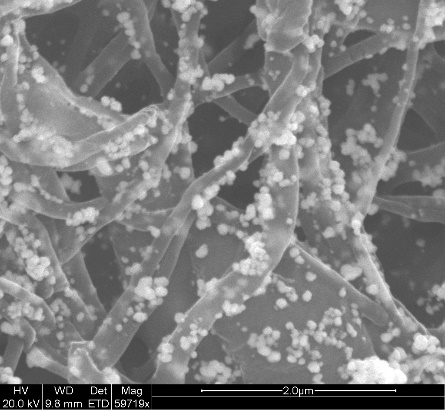

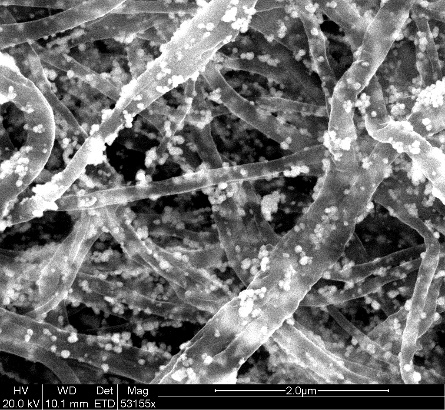

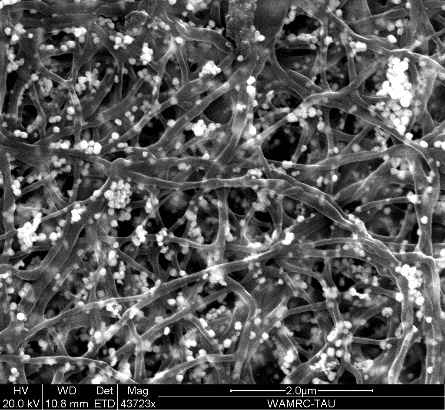

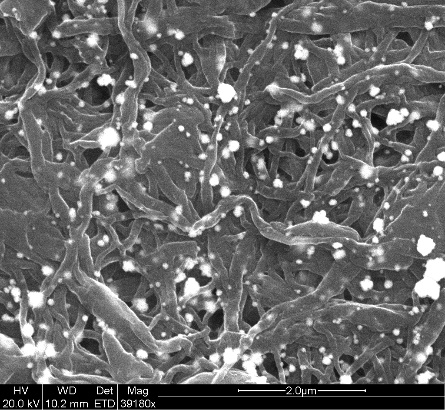


Figure S4. AuNPs synthesized with pH 9 on different JF/PCL NFs scaffolds. (a) 25/75 (b) 33/66 (c) 50/50 (d) 66/33 (e) 75/25

Figure S3. AuNPs synthesized with pH 3 on different JF/PCL NFs scaffolds. (a) 25/75 (b) 33/66 (c) 50/50 (d) 66/33 (e) 75/25


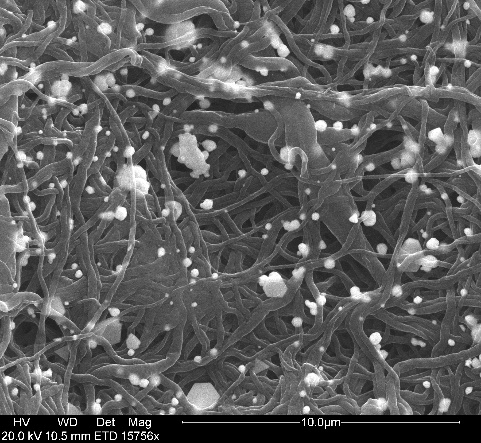

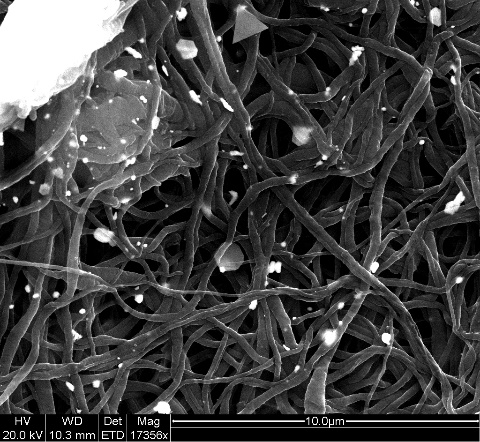

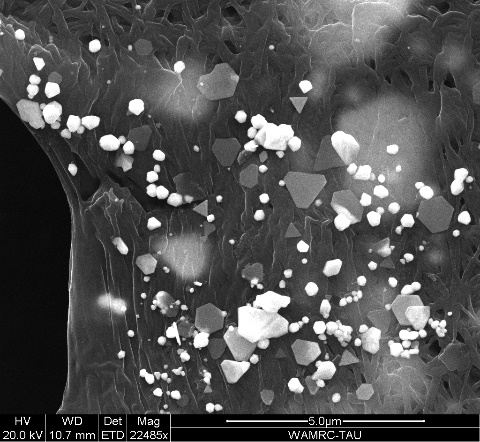

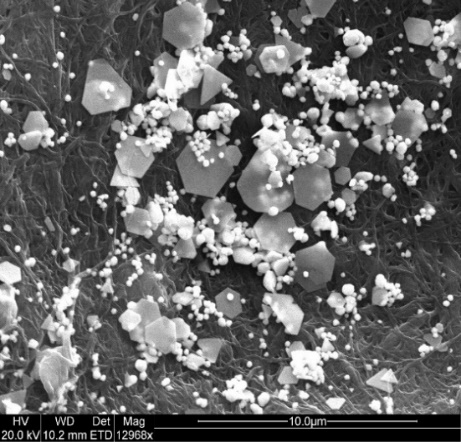

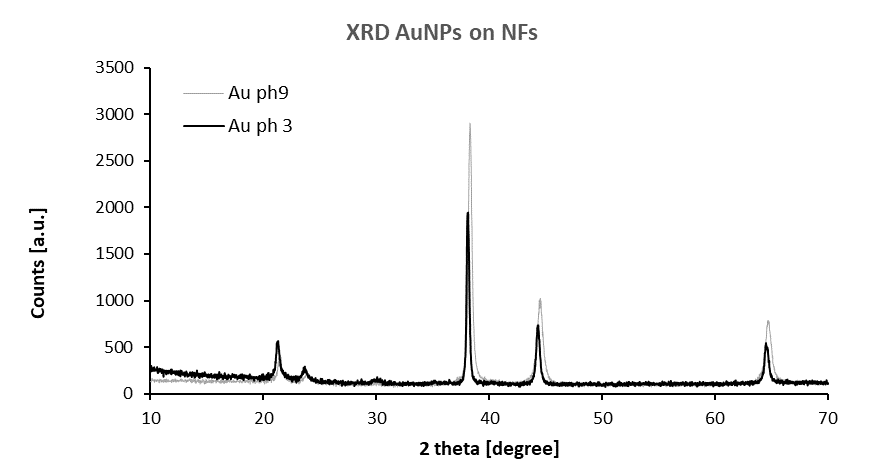

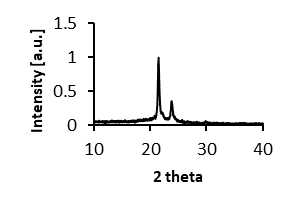


Figure S5. XRD of AuNPs in pH 3 and pH 9. Inset is XRD of pristine JF/PCL scaffold

Figure S6. Particle size dependence on JF/PCL component ratio.


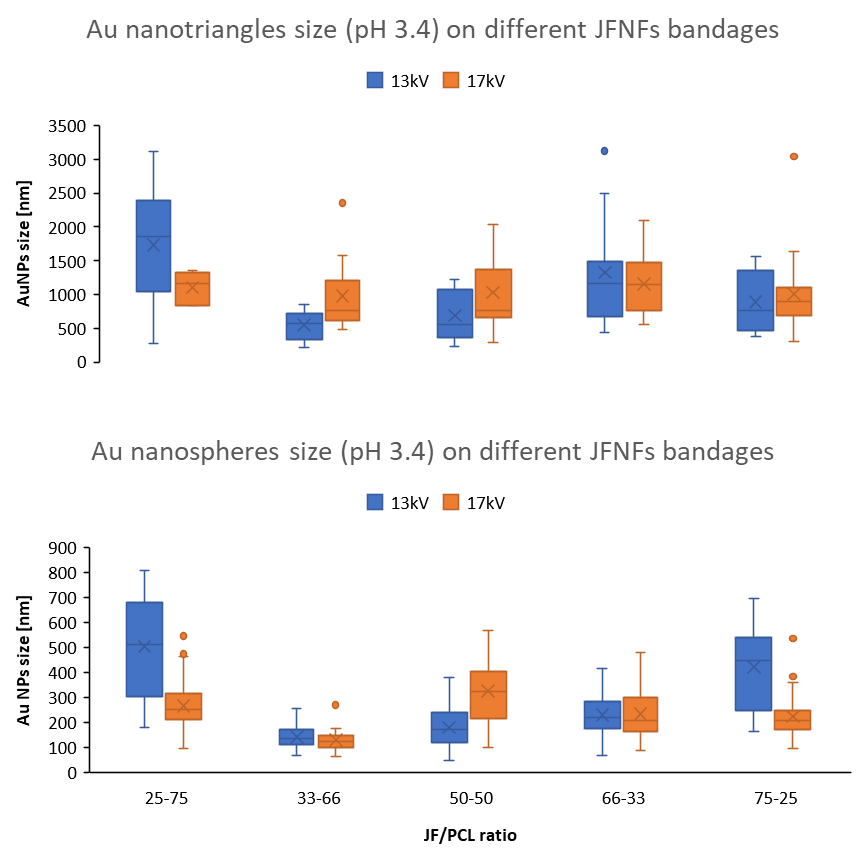

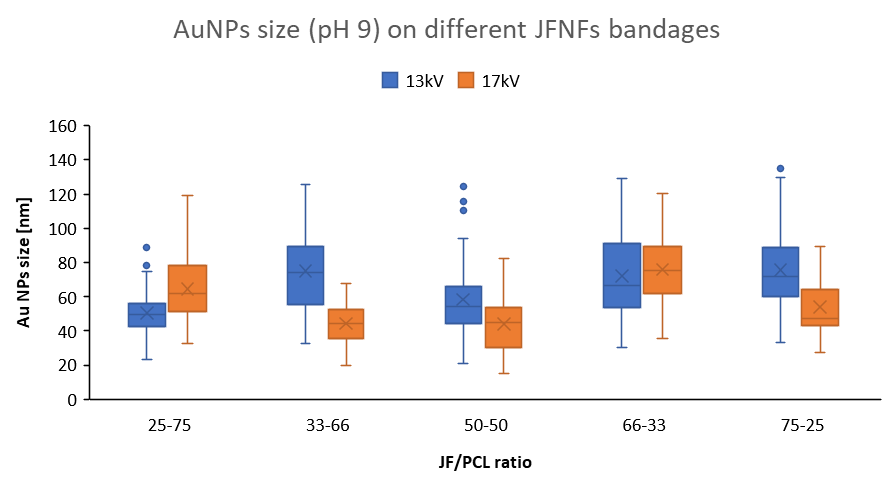


Figure S7. Au-Ag NPs on NFs scaffold under different power output of 808nm laser.


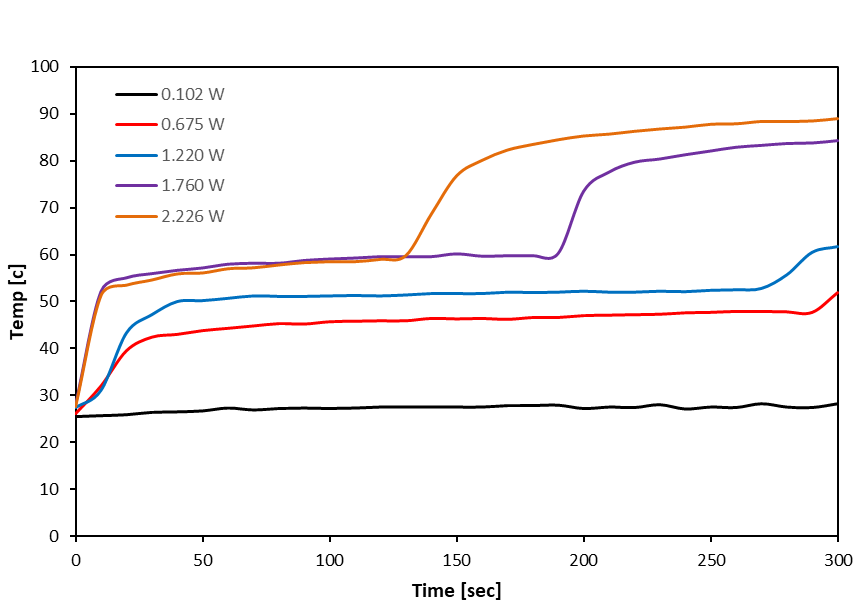


Figure S8. (a) DTA curves of 66/33 and 33/66 JF/PCL scaffolds. (b) TGA curves of 66/33 and 33/66 JF/PCL scaffolds


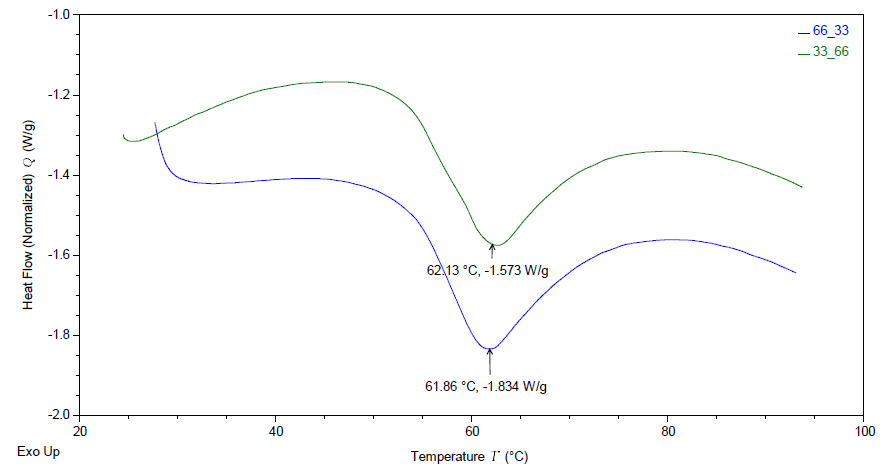

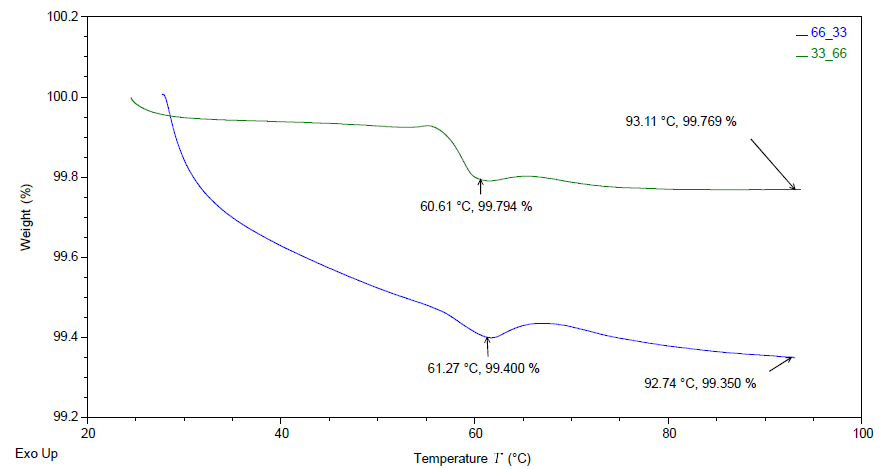


a

b


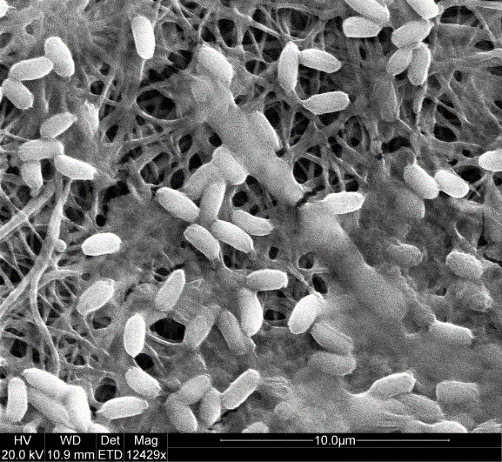

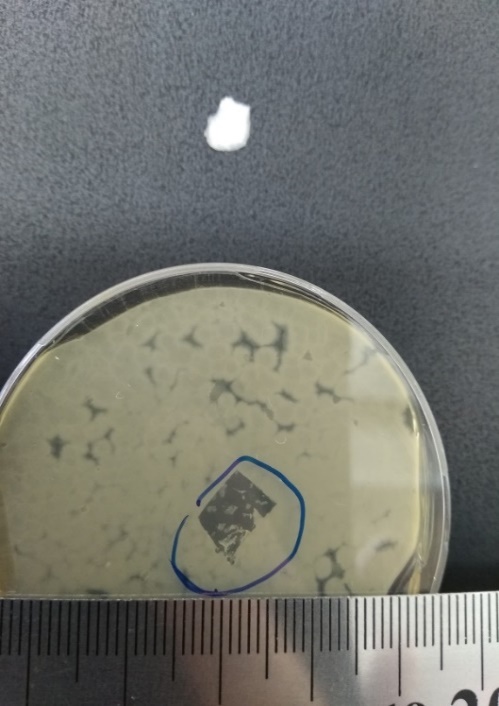


a

b

**Figure S10**. (a) Area underneath pristine NFs scaffold (without NPs) after irradiation with laser for 60 sec. (b) ESEM image of the scaffold after irradiation.

Figure S9. Disk diffusion test of Au NPs pH 9, AgNPs, Au-Ag NPs on NFS scaffolds, after 24 hours on a bacteria culture. (A) Bacteria substrates before scaffolds were removed. (B) bacteria substrates after scaffolds were removed. Au-Ag NPS (NFS scaffolds) that were put on bacteria culture (C) before laser NIR laser irradiation. (D) after 60 seconds of laser irradiation. (G) in area underneath NFS scaffold (50/50 13kv) with Au and Ag after 60 seconds of irradiation


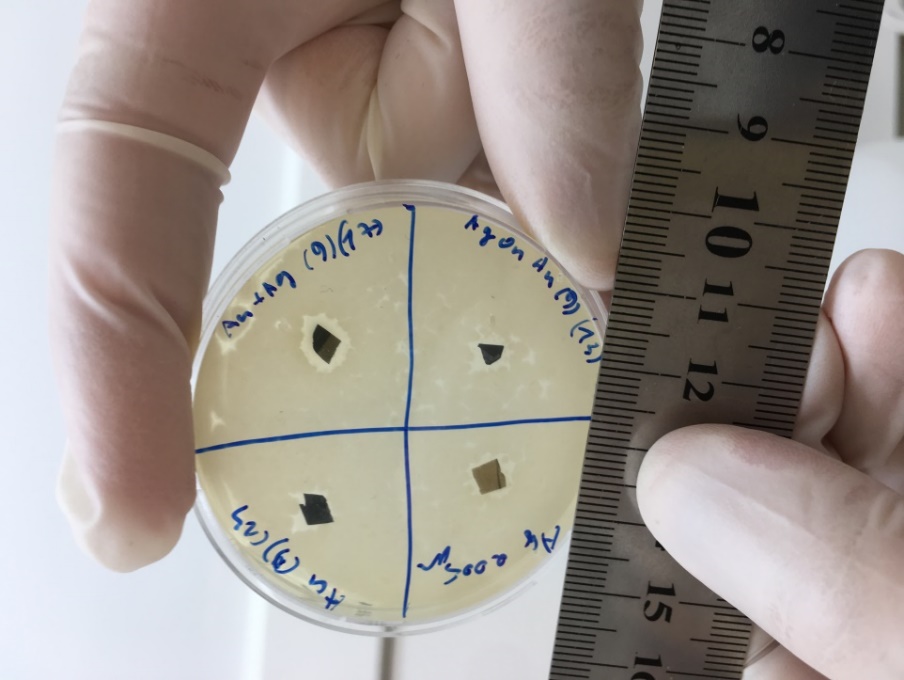

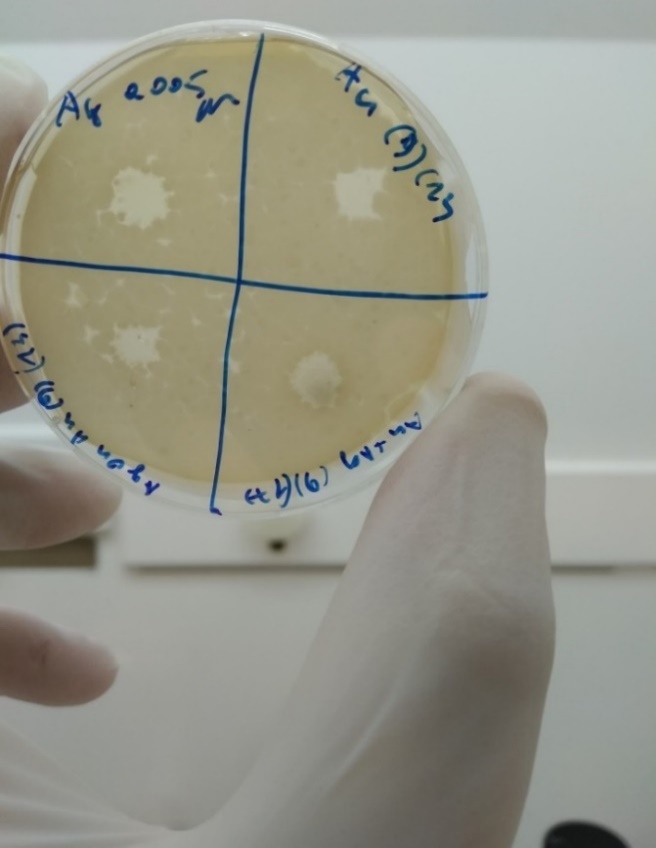


**A**

**B**


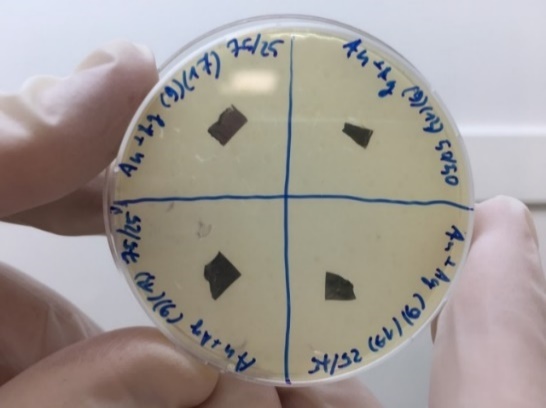

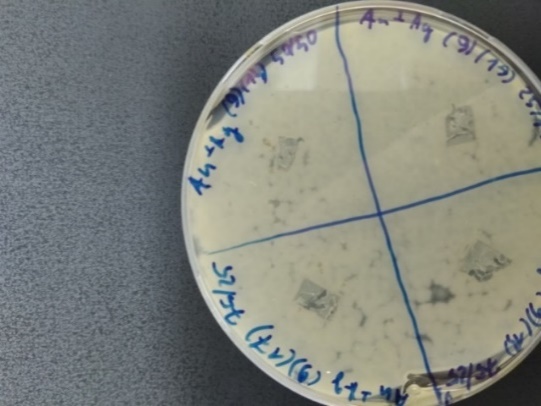


**C**

**D**

**AuNPs pH9**

**Au-AgNPs pH9**

**AgNPs pH9**

**Au-AgNPs pH9**

**Au-AgNPs pH9**

**Au-AgNPs pH9**

**Figure S11. (**a) Swab sample that were taken underneath scaffolds after laser irradiation. (b) Swab sample that were taken underneath the same scaffolds after incubation for 24 hours.


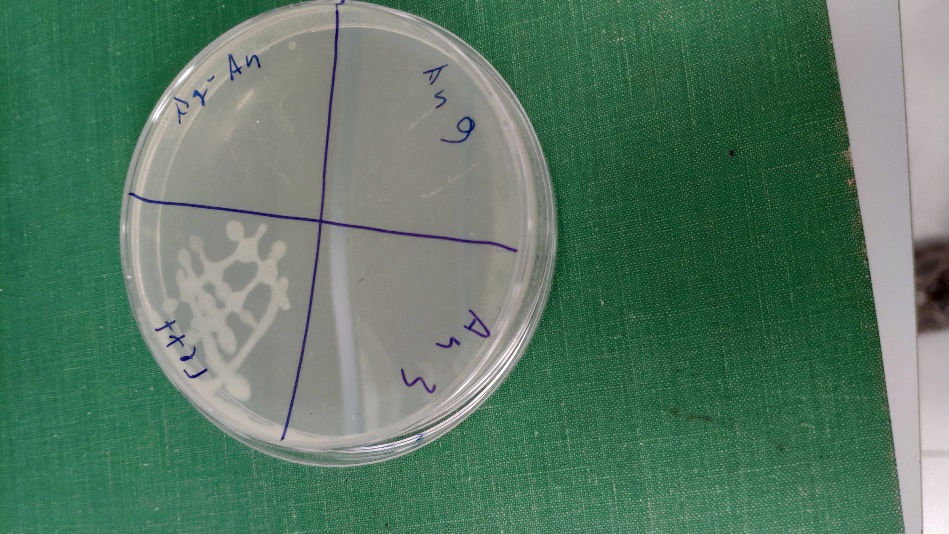

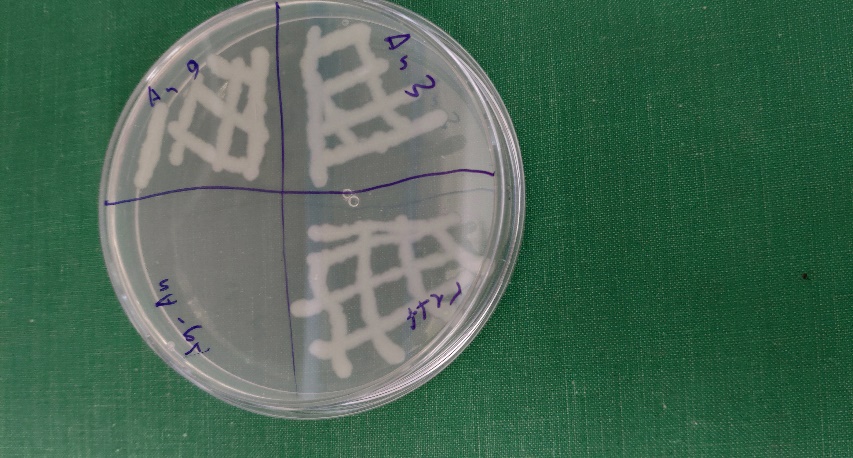


a

b

**
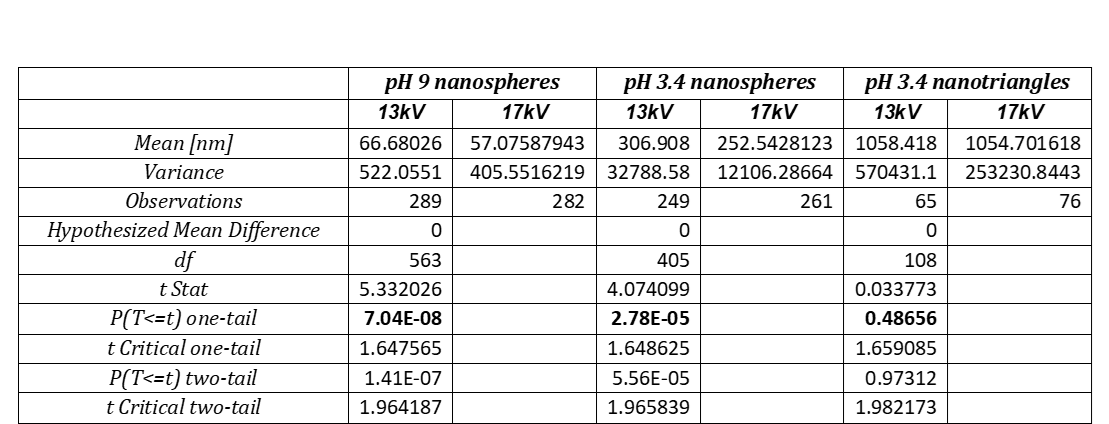
**

Table S1. T-Test for AuNPs size grown in different conditions- pH and JF/PCL scaffold
